# Blood Immune Cell Composition Associated with Obesity and Drug Repositioning Revealed by Epigenetic and Transcriptomic Conjoint Analysis

**DOI:** 10.3389/fphar.2021.714643

**Published:** 2021-10-12

**Authors:** Jia-Chen Liu, Sheng-Hua Liu, Guang Fu, Xiao-Rui Qiu, Run-Dong Jiang, Sheng-Yuan Huang, Li-Yong Zhu, Wei-Zheng Li

**Affiliations:** ^1^ Center of Biomedical Informatics and Genomics, Xiangya Medical College of Central South University, Changsha, China; ^2^ Department of Gastroenterology, The First Affiliated Hospital of University of South, Hengyang, China; ^3^ Department of General Surgery, Third Xiangya Hospital, Central South University, Changsha, China

**Keywords:** obesity, drug repositioning, epigenomics, transcriptomics, immunity, inflammation

## Abstract

This research was designed to analyze the composition of immune cells in obesity and identify novel and potent drugs for obesity management by epigenetic and transcriptomic conjoint analysis. DNA methylation data set (GSE166611) and mRNA expression microarray (GSE18897) were obtained from the Gene Expression Omnibus database. A total of 72 objects (35 obese samples and 37 controls) were included in the study. Immune cell composition analysis, drug repositioning, and gene set enrichment analysis (GSEA) were performed using CIBERSORT, connectivity map (CMap), and GSEA tools. Besides, we performed a single-cell RNA-seq of the immune cells from whole blood samples obtained from one obese patient and one healthy control. mRNA levels of drug target genes were analyzed by qPCR assay in blood samples from six patients and six healthy controls. Immune cell composition analysis found that CD8 + T cells and NK cells were significantly lower in the obese group. 11 drugs/compounds are considered to possess obesity-control potential, such as atorvastatin. Moreover, the expression of drug targets (*STAT3, MCL1, PMAIP1, SOD2, FOX O 3, FOS, FKBP5*) in obese patients were higher than those in controls. In conclusion, immune cells are potential therapeutic targets for obesity. Our results also contribute to accelerate research on drug development of obesity.

## Introduction

Obesity is a chronic metabolic disease caused by the interaction of genetic, environmental, and other factors. Recent data from the 2017–2018 National Health and Nutrition Examination Survey (NHANES) suggest the age-adjusted prevalence of obesity among United States adults was 42.4% and exerted an increasing trend year by year (Fryar and Afful, 2020a; Fryar and Afful, 2020b). As a leading mortality risk factor for Type 2 Diabetes (T2D), coronary heart disease, and other chronic diseases, obesity imposes a considerable economic burden on our medical system and the whole society. For example, a meta-analysis of 97 studies, including 1.8 million participants, suggested that obesity has been linked to an increased risk of coronary heart disease and stroke (Lu et al., 2014). Besides, Epidemiological surveys show that the annual expenditure on obesity and related conditions in the United States is between 147 and 210 billion United States dollars (Cawley and Meyerhoefer, 2012). Therefore, there is an urgent need to address the substantial social burden caused by obesity.

Obesity is a complex disorder caused by the imbalance between energy intake and expenditure (Leeners et al., 2017). Notably, low-grade inflammation is a crucial characteristic of obesity, resulting in the heterogeneity of immune cell composition and polarization (Hotamisligil, 2006; Han and Levings, 2013). Besides, various studies have indicated that obesity-related inflammation is associated with the occurrence of insulin resistance (Kanda et al., 2006; Weisberg et al., 2006), which is a leading risk for T2D and cardiovascular diseases, and the most common and severe complication of obesity (Kochanek et al., 2020).

The continuity and multi-process of obesity make it more lucid to explain the entire development process of obesity at multiple levels (Conway and Rene, 2004; Yan et al., 2018). Notably, multi-omics analysis (Hasin et al., 2017), including epigenomics, transcriptomics, etc., provides an opportunity to better understand the progression of complex diseases (Lee et al., 2020; Yang et al., 2020). For example, a recent study by Zhang et al. (Zhang et al., 2015) which used integration of biological data across genomics, metabolomics, and microbiomics, found that gut microbiota dysbiosis may be an essential cause of genetic and simple obesity in children.

Given the high prevalence rates, economic burden, and complex pathological mechanism, there has been a surge of interest in the effects of obesity pharmacotherapy. The updated guidelines indicated the limitations of existing weight-loss drugs, which suggested an urgent need for drugs with better efficacy and lower prices (Apovian et al., 2015). Due to the high cost and high-level risk of traditional drug development (DiMasi et al., 2010), multi-omics-based drug repositioning may provide a novel approach to solve the problem (Barh et al., 2020). Since the repositioned drugs usually have completed formulation development and even early clinical development (Ashburn and Thor, 2004), their safety in preclinical models and humans is guaranteed (Nosengo, 2016; Pushpakom et al., 2019).

Based on previous research, in this study, we first downloaded the epigenomes and transcriptomes microarray dataset of obesity from the Gene Expression Omnibus (GEO) database. In GSE166611, the study of Nonino et al. comparing methylation patterns between normal weight (*n* = 17) and obese women (*n* = 15) in peripheral blood, which sought to confirm that an obesogenic lifestyle can promote epigenetic changes in the human DNA. In GSE18897, Ghosh et al. (Ghosh et al., 2011) carried out whole-genome expression profiling of whole blood from 60 obese samples and 20 controls. Subsequently, we used EpiDISH and CIBERSORT to reveal the immune cell composition associate with obesity and provide insights into the interplay between host immune response and pathogenesis of obesity. Based on this, drug repositioning screening was performed to identify potential therapeutic agents for obesity, which may shed light in the field of pharmacological intervention of obesity.

## Methods

### Microarray Data Source

The gene methylation profiling datasets (GSE166611) and gene expression profiling datasets (GSE18897), were downloaded from Gene Expression Omnibus (https://www.ncbi.nlm.nih.gov/geo) in the National Center for Biotechnology Information (NCBI). In total, whole blood samples from 20 obese samples and 20 controls were enrolled in GSE18897 (platform: GPL570 Affymetrix Human Genome U133 Plus 2.0 Array). In GSE18897 (GPL13534 Illumina HumanMethylation450 BeadChip), out of 32 peripheral blood samples, 15 were obese, and 17 were normal weight.

### Data Process

DNA methylation microarray data and gene expression profiles data were pre-processed by R (version 4.0.5) and Biomanger packages [limma (Ritchie et al., 2015)], where raw data were background corrected, log-transformed, and quantile normalized. Finally, differentially expressed genes (DEGs) were screened out with *p* < 0.05 and LogFC > 1 as the cut-off criteria.

### Immune Cell Composition Calculation Based on DNA Methylation Data

Immune cell fraction based on DNA methylation data was predicted by EpiDISH algorithm (Teschendorff et al., 2017). The proportion of immune cells between normal samples and obese samples were presented via Bar plots and violin plots, which were all generated in R using ggplot2 package.

### Functional Enrichment Analysis and Pathway Enrichment Analysis

Gene ontology (GO) analysis and Kyoto Encyclopedia of Genes and Genomes (KEGG) pathway enrichment analysis were performed using Hiplot (https://hiplot.com.cn/). GSEA was performed using GSEA 4.1.0 (http://www.broadinstitute.org/gsea).

### Immune Cell Composition Calculation Based on mRNA Expression Profile Data

Immune cells in obese samples was assessed by applying the “Cell type Identification By Estimating Relative Subsets Of RNA Transcripts” (CIBERSORT) deconvolution algorithm (Newman et al., 2015). We set 100 permutations and *p* < 0.05 as the criteria for the successful deconvolution of a sample. Besides, the gene expression signature matrix of six immune cells was obtained from the CIBERSORT platform (https://cibersortx.stanford.edu/).

### Single Cell RNA-Sequencing Analysis

Heparinized blood was mixed with equal volume of PBS buffer (PBS +2% FBS + 2 mm EDTA) and was gently overlaid on density gradient media (Ficoll-Paque Premium 1.073, GE Healthcare, United States) in 15 ml tubes, with volume ratio (blood to media) of 0.75 and Peripheral Blood Mononuclear Cells (PBMCs) were extracted according to manufacturer’s protocol. Remaining red blood cells were removed by red blood cell lysis buffer (Sigma, United States). PBMC were then resuspended in 1 ml freezing media (Fetal Bovine Serum +10% DMSO), and transferred to cryopreservation tubes, and stored in liquid nitrogen.

Frozen cells were thawed and washed by PBS buffer. And Cell concentration was adjusted to around 1,000 cells/ul. 10X Single Cell 3′ V3 kits were used for scRNA-seq library preparation in the same batch following the manufacturer’s protocol (10X Genomics, Pleasanton, CA, United States). We targeted approximately 5,000 cells in each sample and the final libraries were sequenced with pair-end on Illumina platform (Illumina, San Diego, CA, United States), with at least 50,000 reads per cell.

Raw FASTQ data were processed using the 10x Genomics CellRanger pipeline (v3.0.0) to generate a UMI count matrix. After alignment, downstream normalization, scaling and clustering of data were processed using the Seurat package (version 3.0.0) (Fryar and Afful, 2020b) in R (version 3.6.0). Low-quality cells (such as doublets, cells with high expression of mitochondria-associated genes (>20%) were removed. Red blood cells and remaining neutrophils were also removed. t-distributed stochastic neighbor embedding (tSNE) was used for data visualization. The function of FindAllMarkers in Seruat was used to get differentially expressed genes with fold change >0.25 and Bonferroni-adjusted *p* < 0.01 was considered statistically significant.

### Drug Repositioning

Potential drugs for the management of obesity were selected using the Connectivity Map (CMap) database (https:// www.broadinstitute.org/connectivity-map-cmap). CMap is an online tool to identify molecular drugs highly correlated with diseases; the link between the query genes and chemicals was measured with connectivity score ranged from −1 to one and *p* < 0.05. All of the predicted targets were included for the DGIdb and L1000 FWD databases as no threshold was provided. Drug-target and protein-protein interactions among these targets, collected from the STITCH (https://stitch.embl.de/), were used to construct a drug-target interaction network and visualized using Cytoscape v3.8.2.

### RNA Isolation and RT‐PCR Analyses

The total RNA was isolated using RNA extraction kit (TIANGEN) and reverse transcribed into cDNA using reverse transcription kit (ABI). Quantitative real‐time PCR analysis was performed using real‐time PCR kit (ABI). The relative mRNA expression levels of STAT3, MCL1, PMAIP1, SOD2, FOXO3, FOS, and FKBP5were normalized with the GADPH in the same sample. The thermal cycler parameters for the amplification of these genes were as follows: one cycle at 95°C for 10 min followed by 40 cycles at 95°C for 15 s, 60°C for 15 s and 72°C for 30 s. Gene expression was evaluated by the 2−ΔΔCt method. The sequences of RT‐PCR primers are the following (5–3′, Table 1).

**TABLE 1:** The sequences of RT‐PCR primers.

	Forward	Reverse	Amplicon size
STAT3	CAG​CAG​CTT​GAC​ACA​CGG​TA	AAA​CAC​CAA​AGT​GGC​ATG​TGA	150
MCL1	TGC​TTC​GGA​AAC​TGG​ACA​TCA	TAG​CCA​CAA​AGG​CAC​CAA​AAG	135
PMAIP1	ACC​AAG​CCG​GAT​TTG​CGA​TT	ACT​TGC​ACT​TGT​TCC​TCG​TGG	121
SOD2	GGA​AGC​CAT​CAA​ACG​TGA​CTT	CCC​GTT​CCT​TAT​TGA​AAC​CAA​GC	116
FOXO3	CGG​ACA​AAC​GGC​TCA​CTC​T	GGA​CCC​GCA​TGA​ATC​GAC​TAT	150
FOS	GGG​GCA​AGG​TGG​AAC​AGT​TAT	CCG​CTT​GGA​GTG​TAT​CAG​TCA	126
FKBP5	AAT​GGT​GAG​GAA​ACG​CCG​ATG	TCG​AGG​GAA​TTT​TAG​GGA​GAC​T	250
GAPDH	ACA​ACT​TTG​GTA​TCG​TGG​AAG​G	GCC​ATC​ACG​CCA​CAG​TTT​C	—

## Results

### The Identification of Methylated-Differentially Genes and the Composition of Immune Cells in Obesity

**FIGURE 1:**
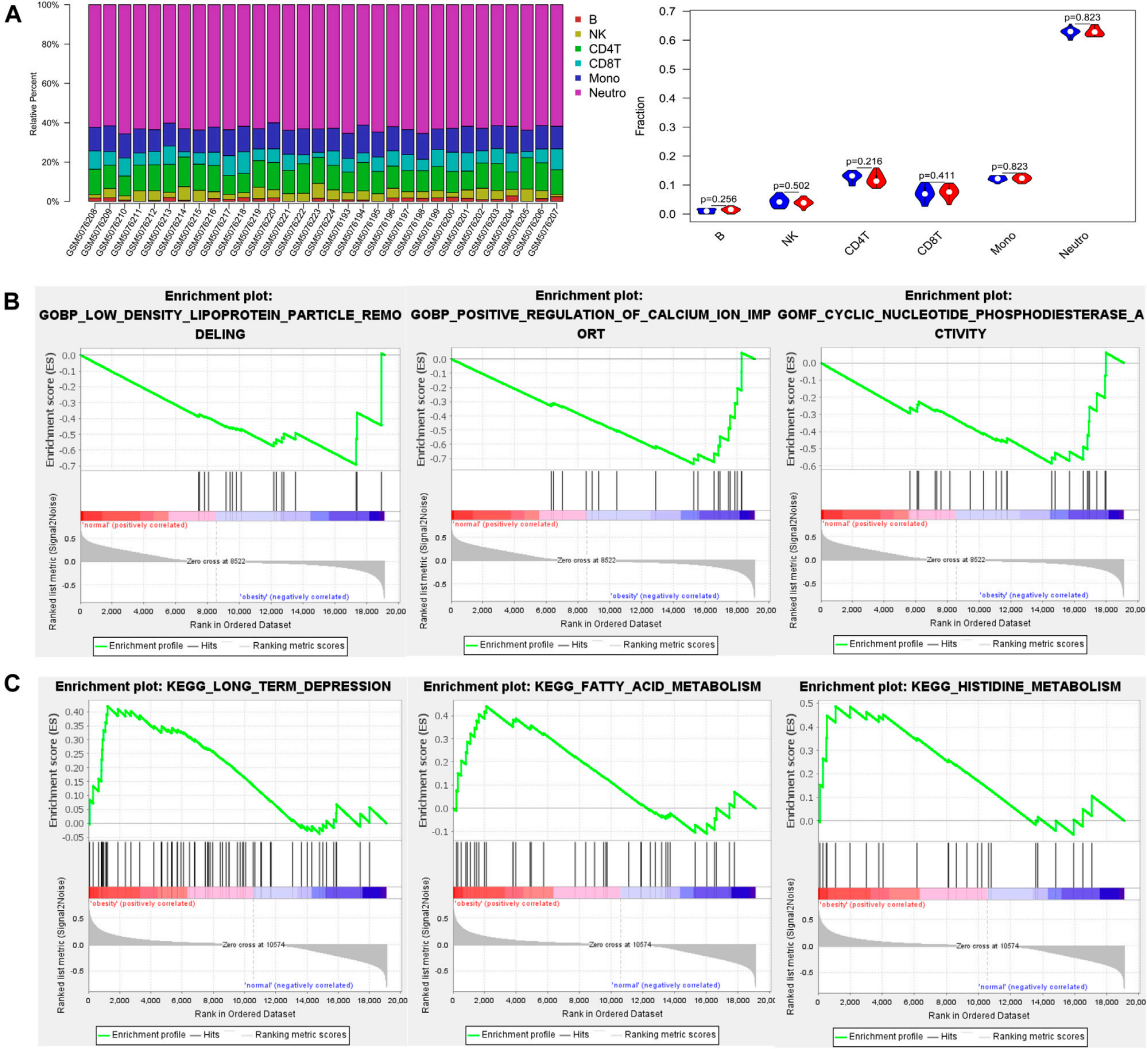
The proportion of immune cells and gene set enrichment analysis (GSEA) results based on DNA methylation profiles between normal and obese samples **(A)** The proportion of immune cells between normal and obese samples. The quantified contrast of the distribution of obesity‐related immune cell subtypes and the difference in immune composition in each obesity and control tissues sample **(B)** Enrichment plots from gene set enrichment analysis (GSEA). The low-density lipoprotein particle remodeling, positive regulation of calcium ion import, and cyclic nucleotide phosphodiesterase activity genes were significantly enriched in obesity groups **(C)** Clustering of KEGG pathways identified by GSEA analysis. Pathway enrichment analysis revealed a variety of signaling pathways in which fatty acid metabolism and histidine metabolism may be relevant to the pathology of obesity.

Analysis of the methylation profiles by the EpiDISH algorithm was used to estimate the proportion of obesity-related immune cell composition. There was no significant difference in the immune cell composition between normal and obese populations (Figure 1A).

Then, we conducted gene set enrichment analysis (GSEA) to determine the biological processes modulated by DNA methylation in obese patients. The results showed the low-density lipoprotein particle remodeling, positive regulation of calcium ion import, and cyclic nucleotide phosphodiesterase activity genes were significantly enriched in the obesity groups (Figure 1B). Besides, pathway enrichment analysis revealed a variety of signaling pathways in which fatty acid metabolism and histidine metabolism may be relevant to the pathology of obesity (Figure 1C).

### The Immune Cell Composition Status From Transcriptomes of the Obese Cohort

**FIGURE 2:**
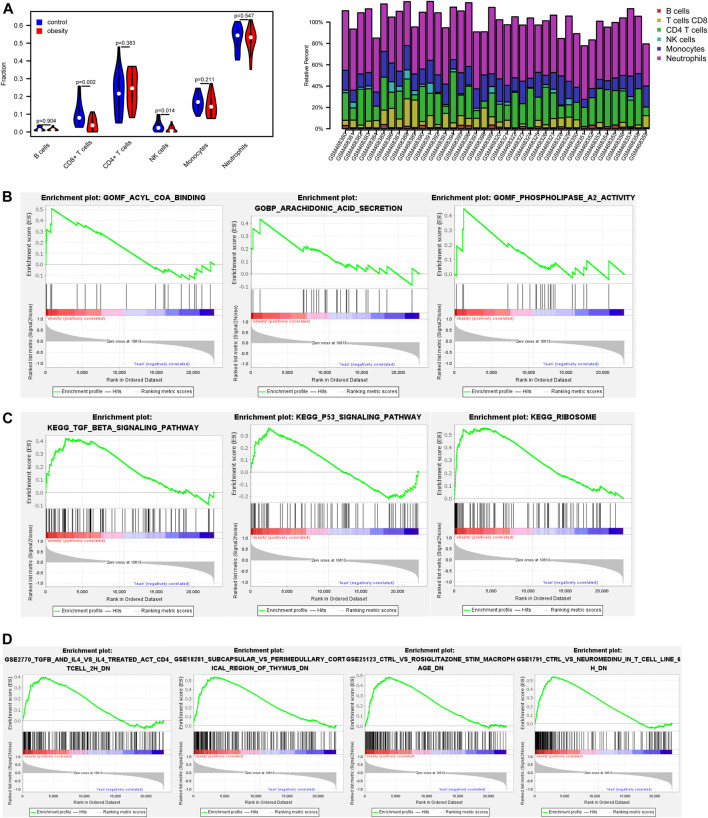
The proportion of immune cells and gene set enrichment analysis (GSEA) results based on gene expression profiles between normal and obese samples **(A)** The proportion of immune cells between normal and obese samples. Among these, CD8 + T cells and NK cells were negatively associated with obesity **(B)** Enrichment plots from gene set enrichment analysis (GSEA). The arachidonic acid secretion, acyl CoA binding, and phospholipase a2 activity were significantly enriched in obesity groups **(C)** Clustering of KEGG pathways identified by GSEA analysis. Pathway enrichment analysis revealed TGF-β signaling pathway and p53 signaling pathway may be relevant to the pathology of obesity **(D)** Enrichment of immune signatures by GSEA analysis. Immunologic signatures most correlated with NeuromedinU, TGF-β, IL-4 in obese patients.

The immune composition in obese and control was analyzed by the CIBERSORT algorithm (Figure 2A). The results showed that the proportion of immune cells was different in each subgroup. Among these, CD8 + T cells and NK cells was significantly lower in the obese group.

GSEA results showed that arachidonic acid secretion, acyl CoA binding, and phospholipase a2 activity were closely correlated with obesity (Figure 2B). Pathway enrichment analysis revealed significant enrichment for the TGF-β signaling pathway and p53 signaling pathway (Figure 2C). Besides, enrichment of immune signatures was further analyzed using GSEA, and results showed that immunologic signatures most correlated with NeuromedinU, TGF-β, IL-4 (Figure 2D).

### Enrichment Analysis of Immune Cells Using Single-Cell RNA-Sequencing Reference Data

**FIGURE 3:**
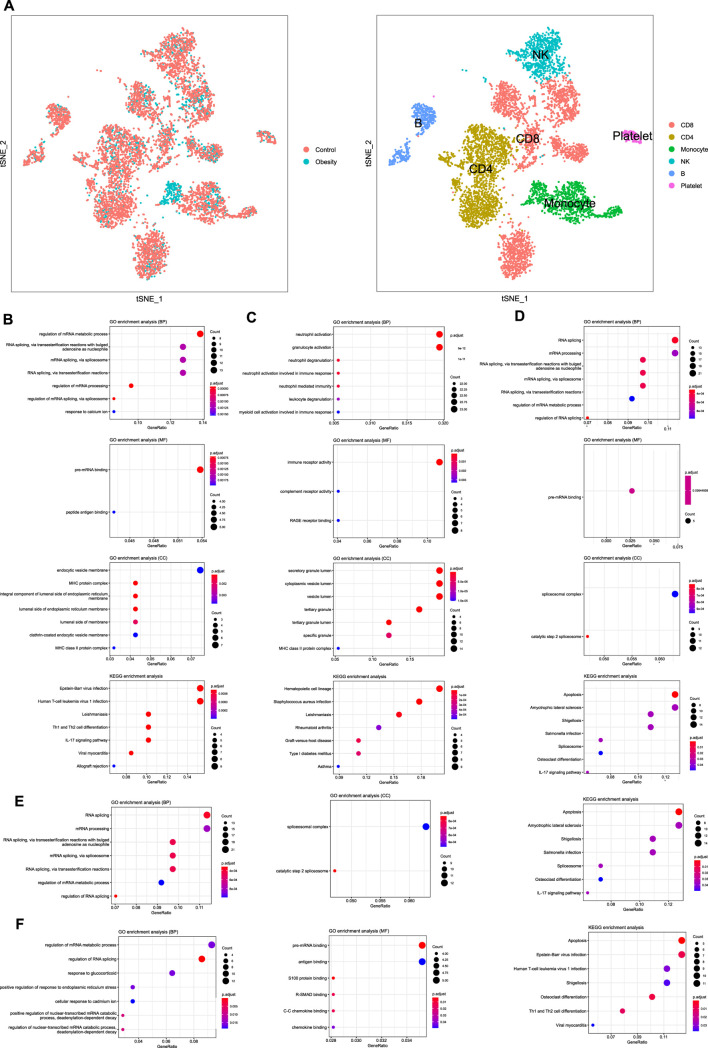
Single-cell RNA sequencing and enrichment analysis of immune cells **(A)** TSNE plot of PBMCs from different groups and different cell type **(B)** Enrichment analysis of B cells **(C)**Enrichment analysis of CD8 + T cells **(D)** Enrichment analysis of CD4 + T cells **(E)** Enrichment analysis of NK cells **(F)** Enrichment analysis of monocytes.

We performed single-cell sequencing of one obese patient and one healthy control. Enrichment analysis of B cells (Figure 3A), CD8 + T cells (Figure 3B), CD4 + T cells (Figure 3C), NK cells (Figure 3D), Monocytes (Figure 3E) was conducted. The enrichment analysis revealed that some common GO terms, such as MHC protein complex and response to calcium ion, were significantly enriched in various immune cell types. Besides, according to the annotation of KEGG, apoptosis and IL-17 signaling pathway were enriched in NK cells and CD4 + cells, which were negatively associated with obesity based on the above findings.

### Drug Repurposing Based on Gene Expression Profile

#### Identification of Expression Profiles of Obese Patients

A total of 204 obesity-associated genes, including 173 up-regulated genes and 31 down-regulated genes (*p*-value < 0.05, logFC >1) were extracted from mRNA profiles of 20 obese patients and 20 controls.

#### Obesity-Targeted Screening for Candidate Drugs

**FIGURE 4:**
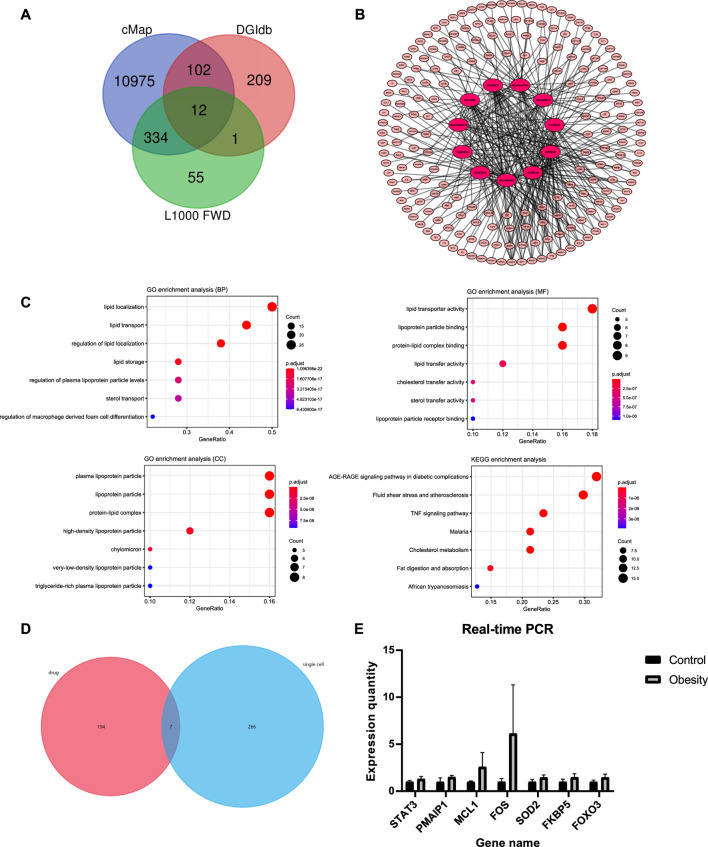
Drug repositioning and network pharmacology analysis **(A)** The screening results in CMap, DGIdb, and L1000 FWD databases; 12 repeated compounds were shared among all three databases **(B)** Pharmacology-network of the “drugs-targets-disease”; The red ellipse represent drugs. The pink ellipse represents drug targets of genes **(C)** GO and KEGG analyses of target genes of atorvastatin **(D)** Screening of the hub genes; seven genes among 201 drug targets (*STAT3, MCL1, PMAIP1, SOD2, FOX O 3, FOS, FKBP5*) were overlapping with differential genes from single-cell sequencing **(E)** Quantitative real-time PCR analysis of hub genes (n = 2 for each group); The expression of the corresponding mRNAs was all up-regulated in obese patient.

According to the above results, a total of 11,675 compounds were screened from the CMap, DGIdb and L1000 FWD databases (Figure 4A). The overlapping 12 compounds among the three databases were considered as potential drugs of obesity (Table 2).

**TABLE 2:** Eleven chemicals were identified as potential interventions for obesity.

Generic name	Associated conditions	Adverse effects	Price
Daunorubicin	Acute Lymphocytic Leukemia Acute Myeloid Leukemia Acute erythroid leukemia Acute monocytic leukemia Newly diagnosed Therapy-Related Acute Myeloid Leukemia	Sores Trouble swallowing Dry mouth Bad breath Altered sense of taste Nausea	163.01 USD/4 ml
Menadione	Factor II deficiency Vitamin B12 Deficiency	Hemolytic anemia Brain damage	5.59 USD/5 mg
Docetaxel	Esophageal Cancers Ewing’s Sarcoma Locally Advanced Breast Cancer Metastatic Bladder Cancer Metastatic Breast Cancer Metastatic Hormone Refractory Prostate Cancer Node Positive Breast Cancer Ovarian Cancer Metastatic Small Cell Lung Cancer	Black, tarry stools Bleeding gums Blood in the urine or stools Chest pain Chills Diarrhea Heartburn Dizziness Hoarseness Irritation	477.37 USD/0.5 vial
Alvocidib (Flavopiridol)	Esophageal cancer Leukemia Lung cancer Liver cancer	Nausea Fatigue Hepatic dysfunction Myelosuppression	11.4 USD/mg
Staurosporine	Acute Myeloid Leukemia (AML) Aggressive Systemic Mastocytosis Systemic Mastocytosis with Associated Hematological Neoplasm	Abdominal or stomach cramps or pain Accumulation of pus Backache Nasal congestion Nausea	156.1 USD/mg
Rucaparib	Advanced Ovarian Cancer Fallopian tube cancer Prostate cancer with an abnormal BRCA gene	Low blood cell counts Shortness of breath Cold symptoms such as stuffy nose, sneezing, Sore throat Stomach pain Nausea Vomiting	21.4 USD/mg
Cladribine	Chronic Lymphocytic Leukaemia Cutaneous T-Cell Lymphoma Hairy Cell Leukemia Non-Hodgkin's Lymphoma	Hives Difficult breathing Headache Fever Unusual bleeding Night sweats	102.78 USD/10 mg
Atorvastatin	Anginal Pain Cardiovascular Disease Coronary Artery Disease Coronary artery thrombosis Dysbetalipoproteinemia Fredrickson Type III lipidemia High Cholesterol Hospitalizations Hypertriglyceridemias Postoperative Thromboembolism Primary Hypercholesterolemia	Joint pain Stuffy nose Sore throat Diarrhea	Tablet: 5.0 USD/20 mg
Irinotecan	Esophageal Cancers Ewing’s Sarcoma Glioblastomas Malignant Neoplasm of Pancreas Malignant Neoplasm of Stomach Metastatic Colorectal Carcinoma Non-Small Cell Lung Carcinoma Ovarian Cancer Rhabdomyosarcomas Small Cell Lung Cancer	Fever Mouth sores Low blood cell counts Diarrhea Constipation Nausea Vomiting Stomach pain Loss of appetite Weight loss	138.07 USD/2 ml
Erlotinib	Locally Advanced Non-Small Cell Lung Cancer Locally Advanced Pancreatic Cancer Metastatic Non-Small Cell Lung Cancer Non-Small Cell Lung Carcinoma Pancreatic Cancer Metastatic	Burning, tingling, numbness or pain in the hands, arms, feet, or legs Cough Hoarseness Diarrhea (severe) Difficult or labored breathing Fever or chills Lower back or side pain Rash (severe) Sensation of pins and needles Stabbing chest pain	Tablet: 163.98 USD/150 mg
Sorafenib	Advanced Renal Cell Carcinoma Gastrointestinal Stromal Tumors Hemangiosarcoma Unresectable Hepatocellular Carcinoma Locally recurrent refractory to radioactive iodine treatment Thyroid carcinoma Metastatic refractory to radioactive iodine treatment	Bleeding Feeling tired Vomiting Diarrhea Nausea Stomach pain High blood pressure Rash Weight loss Thinning hair	Tablet: 66.61 USD/200 mg

### Network Pharmacology Approach to Predicting the Mechanisms of Drugs Counteracts Obesity

#### The Collection of Pathogenic-Related Genes of Obesity

We comprehensively collected the genes associated with obesity from Online Mendelian Inheritance in Man (OMIM) database, GeneCards, and DisGeNET. Based on this, we created a final list of 2,316 pathogenic-related genes of obesity that were reported in at least two examined databases.

### Identification of Drug Targets and Construction of the Drug-Targets Network

A total of 466 genes related to 11 compounds were identified using STITCH prediction (One of the compounds was excluded from the drug candidates owing to a smaller number of targets.) Among these, 201 genes belonged to pathogenic-related genes, as mentioned above. Finally, a network was constructed to display the association between the gene targets and candidate drugs (Figure 4B). Notably, atorvastatin was considered the most likely candidate for the treatment of obesity as 43/50 of gene targets were overlapping with pathogenic-related genes. Besides, through consulting literature, atorvastatin is closely related to the progress of obesity. Therefore, the target genes of atorvastatin were further analyzed by GO and KEGG analyses (Figure 4C). The results show that atorvastatin may affect the redistribution of lipids, thereby influencing the development of obesity.

### Screening and Identification of the Hub Genes

To further examine the drug targets, we focused on 201 drug targets and find that seven genes, *STAT3, MCL1, PMAIP1, SOD2, FOXO3, FOS, FKBP5* were overlapping with differential genes from single-cell sequencing (Figure 4D). Expression of selected genes was subjected to real-time quantitative PCR (RT-qPCR) verification from six obese patients and six controls, which revealed that the expression of the corresponding mRNAs was all up-regulated in obese patients (Figure 4E).

## Discussion

Methylation signals in the blood may serve as surrogate markers for immune cells and provide molecular insight into immune-related diseases such as obesity. In this study, we performed GSEA to determine the different biological functional states of obese and normal samples. In the obesity group, the enriched GO terms were mainly focused on low-density lipoprotein (LDL) particle remodeling, cyclic nucleotide phosphodiesterase activity, and positive regulation of calcium ion import. The result is consistent with those of Roderick FDJ et al. (King et al., 2008), which compared the phenotype and composition changes of LDL-c particles before and after the weight loss of nine obese children. They found that the LDL-c III particles and their relative cholesterol concentration decreased significantly, while the proportion of type II and type I particles increased. In addition, a study by Xue et al. (Xue et al., 2001) indicated that through activating phosphodiesterase activity to promote the degradation of cAMP, calcium ions in fat cells could reduce the phosphorylation of hormone-sensitive lipase, thereby inhibiting lipolysis. Based on the KEGG database, we found the enrichment in various metabolic pathways in the obesity group, such as fatty acid metabolism and histidine metabolism. Many studies have proved that the increase of free fatty acids can enhance insulin resistance and increase the expression of pro-inflammatory cytokines to aggravate obesity (Boden et al., 2002; Itani et al., 2002). Moreover, a vicious cycle may be created by increasing plasma-free fatty acids, which can inhibit the anti-lipolytic effect of insulin and promote the release of free fatty acids (Jensen et al., 1989). Besides, an imbalance in the ratio of n-6/n-3 polyunsaturated fatty acids (PUFAs) may lead to adipose tissue hyperplasia (Ailhaud et al., 2008). Similarly, there is evidence that a diet high in omega-6 fatty acids during the perinatal period would cause accumulation of body fat in offspring (Massiera et al., 2010). On the other hand, histidine is an essential amino acid, which proper supplementation has a positive effect on resisting obesity-related inflammation. Animal-based studies have shown that supplementing histidine can significantly increase the expression of the anti-inflammatory factor adiponectin by activating PPARg. Additionally, another anti-inflammatory mechanism of histidine is manifested in its inhibition of the transfer of the p65 subunit of NF-kB to the nucleus, which can reduce the stimulation of IL-6 and other pro-inflammatory factors, thereby improving the inflammation of obesity (Sun et al., 2014). Thus, histidine metabolism disorder may be associated with the progression of obesity-related inflammation, which is worth exploring.

In the present study, we aimed to clarify potential pathways of obesity development through analyzing transcriptomics data. The GO terms were enriched in arachidonic acid secretion, Acyl CoA binding, and phospholipase A2 activity. Interestingly, they seem to be included in the same story. Acyl-CoA synthase can indirectly enhance the uptake of exogenous arachidonic acid (DiRusso and Black, 1999). The endogenous arachidonic acid is mainly released from cell membrane phospholipids. This process is catalyzed by the phospholipase A2 (PLA2) superfamily of enzymes and is induced by various cell activation signals, including inflammatory stimuli and so on (Dennis et al., 2011). Corresponding to the foregoing, arachidonic acid is an omega-6 polyunsaturated fatty acid, and the instability of its secretion may lead to the massive production of pro-inflammatory eicosanoids and thus aggravate the state of obesity (Saini and Keum, 2018). In the current study, the information of metabolic pathways obtained from the KEGG database revealed that the TGF-β signaling pathway, p53 signaling pathway, and ribosome are enriched in the obese cohort. A recent study by Hariom et al. (Yadav et al., 2011) suggested that blocking the TGF-β/Smad3 pathway would reduce fat mass by down-regulating the expression of adipocyte-specific genes and reducing the infiltration of pro-inflammatory macrophages. Besides, Shjie et al. (Liu et al., 2017) found that the expression of c-MYC was increased in high-fat-fed mice whose RPL11 binding was disrupted, thereby up-regulating the biogenesis of ribosomes and promoting nutrient absorption through the RPL11-MDM2-p53 pathway, resulting in obesity-related metabolic changes. Besides, the composition of immune cells might be related to the state of obesity. The results of GSEA based on immunity and immunology showed that the level and status of immune cells such as macrophages, CD4 + T cells, CD8 + T cells, and B cells have changed during the progress of obesity, which also involves cytokines such as IL-4.

One interesting finding is the immune cell composition status from transcriptomes of the obese cohort. The results showed a significant decrease in CD8 + T cells and NK cells in the obese group compared with the control group. This finding is different from the accepted conclusion that the infiltration of CD8 + T cells and NK cells in the adipose tissue of obese patients is increased (Chatzigeorgiou et al., 2012). The possible reason is the samples we analyzed are blood samples instead of adipose tissue, which may have lost the activation effect of adipose tissue-derived factors on the relevant immune cells.

The results of single-cell sequencing reflected the enrichment of the differentially expressed genes of the five immune cells based on the GO and KEGG databases. The result revealed that B cells is mainly implicated in MHC protein complex, endocytic vesicle membrane, and clathrin-coated endocytic vesicle membrane. The binding of the antigen to the B cell receptor (BCR) initiates B cell activation, triggers BCR endocytosis, and recruits peripheral vesicles containing MHC class II to the lysosomal-like antigen processing chamber containing the antigen BCR. The internalized antigens are processed into peptides in these compartments and produce peptide-MHC class II complexes (Raposo et al., 1996), transported to the plasma membrane for recognition by CD4 + T cells for subsequent immune response (Lankar et al., 2002). In addition, the process by which IL-23 can stimulate CD4 + T cells to secrete IL-17 pro-inflammatory factors has been confirmed (Harrington et al., 2005), which corresponds to the result of the IL-17 signaling pathway in the KEGG pathway enrichment. Besides, apoptosis is significantly enriched in CD8 + and CD4 + T cells and NK cells. Fas and Fas ligand (FasL) induce apoptosis of immune cells (Restifo, 2000), which are rapidly expressed after the TCR/CD3 complex of primary T cells is activated by the peptide antigen presented by MHC class II or MHC class I. The data taken from Alderson et al. showed that activated CD8 + and CD4 + T cells express high amounts of FasL and are susceptible to Fas antibody-mediated apoptosis, thereby limiting the expansion of the immune response (Alderson et al., 1995). Thus, the results suggested that apoptosis may play a significant role in maintaining immune homeostasis and reducing inflammation in obese patients, which needs future studies to improve knowledge. Furthermore, the enrichment results of KEGG also indicated an association among osteoclast differentiation and CD4 + and CD8 + T cells. A previous study provides strong evidence for this result, which proves that similar to the role of B cells, osteoclasts are professional antigen-presenting cells (APCs), which can present antigens through MHC protein complex and activate CD4 + and CD8 + T cells (Li et al., 2010). Conversely, it seems that CD8 + T cells can inhibit the production of cytokines that stimulate osteoclast differentiation by producing certain inhibitors (John et al., 1996). The complex feedforward and feedback mechanisms between osteoclasts and CD4 + and CD8 + T cells may be one reason for the number of T cells and the secretion of related cytokines.

Furthermore, the results of qPCR analysis validated our bioinformatic analyses that the expression of selected genes was all up-regulated, which significantly reflects the complex regulation of the inflammatory state in obese patients. Recalling previous animal studies that identified macrophage-intrinsic miR-17–92 family miRNAs promote FOS expression by inhibiting the YY1 protein, thereby maintaining the optimum expression of the anti-inflammatory cytokine IL-10 (Zhang et al., 2020). Moreover,a study of animal models suggested that the differentiation of Th17 cells and the production of IL-17 of $Batf^{-/-}$ mice with normal expression of AP-1 transcription factor composed of Jun, Fos, and MAF was inhibited. It provided evidence that FOS has an inhibitory effect on the secretion of pro-inflammatory factors by CD4 + T cells (Schraml et al., 2009). Thus, the high expression of FOS in obese patients may be negative feedback to persistent low-grade inflammation. In the present study, as expected, the increased expression of STAT3 in the obese group promotes obesity through the IL-23/STAT3/Th17 axis. The primary downstream molecule of the IL-23 signal pathway is STAT3 (Parham et al., 2002), which can facilitate the development of Th17 cells by activating RORC, thereby promoting the release of pro-inflammatory cytokines such as IL-17 to induce inflammation (Isono et al., 2014). Besides, since IL-27 can activate STAT3, STAT3 also appears to be contained in the IL-27-mediated up-regulation of IL-10 (Stumhofer et al., 2007).

Due to the lack of effective/safe and less expensive drugs, drug repositioning appears to be the best tool for finding proper targets and predicting latent drugs in the therapy for obesity and related complications. Finally, 12 compounds were predicted to have potential therapeutic effects on obesity (Table 2) Among these, atorvastatin is a class of cholesterol-lowering drugs that inhibit the enzyme 3-hydroxy-3-methylgluteryl CoA reductase, which are the first choices for treating the hyperlipidemia (Shitara and Sugiyama, 2006). In addition, animal-based studies have shown that atorvastatin can restrain adipose tissue inflammation by inhibiting the activation of nuclear factor-κB and its activation inducers (Furuya et al., 2010; Yamada et al., 2017), indicating its pleiotropic effect on obesity control. Furthermore, its low price among these therapeutic agents makes it more conducive to being accepted by patients. As reported in a previous study concerning the effect of alvocidib on preventing inflammation, alvocidib could block the infiltration of leukocytes and their interaction with endothelial cells by preventing the activation of endothelial cells (Schmerwitz et al., 2011).

Concerning the research methods, some limitations need to be acknowledged. One of them was the lack of detection indicators or the small sample size, which may lead to the high probability of negative results in differential DNA methylation. Besides, the limitations of algorithms made an overall conclusion about the relationship between immune cell composition extremely difficult. In addition, the drugs we have found are conclusions based on systems biology methods, which are needed to be validated by animal models or cohort studies in the future. Notwithstanding these limitations, this work offers valuable insights into exploring inflammation during obesity through a multi-omics approach and developing novel clinical management of obesity.

## Conclusion

The principal findings of this research are that CD8 + T cells and NK cells were significantly lower in the obese group calculated by CIBERSORT. 12 drugs/compounds are considered to possess obesity-control potential, such as atorvastatin. Moreover, *STAT3, MCL1, PMAIP1, SOD2, FOXO3, FOS, FKBP5* may play an essential role in the genesis and growth of obesity and might serve as a possible therapeutic target.

## Data Availability

The original contributions presented in the study are publicly available. This data can be found here: https://www.ncbi.nlm.nih.gov/geo/query/acc.cgi?acc=GSE166611 and https://www.ncbi.nlm.nih.gov/geo/query/acc.cgi?acc=GSE18897
