# Corrigendum: Blood Immune Cell Composition Associated With Obesity and Drug Repositioning Revealed by Epigenetic and Transcriptomic Conjoint Analysis

**DOI:** 10.3389/fphar.2021.838902

**Published:** 2022-01-10

**Authors:** Jia-Chen Liu, Sheng-Hua Liu, Guang Fu, Xiao-Rui Qiu, Run-Dong Jiang, Sheng-Yuan Huang, Li-Yong Zhu, Wei-Zheng Li

**Affiliations:** ^1^ Xiangya Medical College of Central South University, Changsha, China; ^2^ Department of Gastroenterology, The First Affiliated Hospital of University of South, Changsha, China; ^3^ Department of General Surgery, Third Xiangya Hospital, Central South University, Changsha, China

**Keywords:** obesity, inflammation, immunity, transcriptomics, epigenomics, drug repositioning

An author name was incorrectly spelled as Yong-Li Zhu. The correct spelling is Li-Yong Zhu.

The authors apologize for this error and state that this does not change the scientific conclusions of the article in any way. The original article has been updated.

